# CRISPR Genome Editing and the Future of Leukaemia Immunotherapy

**DOI:** 10.1002/hsr2.72497

**Published:** 2026-05-06

**Authors:** Dejin Rai, Umberto Terranova

**Affiliations:** ^1^ Faculty of Medicine and Health Science University of Buckingham Buckingham UK

**Keywords:** base editing, CRISPR‐Cas9, gene editing, immunotherapy, leukaemia

## Abstract

**Background and Aims:**

Leukaemia presents ongoing therapeutic challenges due to relapse and toxicity associated with standard treatments. By enabling more targeted and safer therapies, CRISPR genome editing is emerging as a powerful tool to address these issues.

**Methods:**

We review current literature on CRISPR technologies in leukaemia immunotherapy, focussing on studies involving four key antigens commonly targeted in leukaemia: CD33, CD7, CD45 and CD19.

**Results:**

We trace the evolution of CRISPR technologies from conventional CRISPR‐Cas9 to base editing, highlighting how CRISPR platforms are being repurposed to enhance efficacy and clinical safety. Key studies targeting CD33 demonstrate how editing strategies can enable safer acute myeloid leukaemia (AML) therapies by reducing off‐tumour toxicity; those addressing CD7 show how base editing prevents T‐cell fratricide in T‐cell acute lymphoblastic leukaemia (T‐ALL) immunotherapy; studies focusing on CD45 illustrate how targeted editing facilitates universal CAR T‐cell therapy; and clinical trials on CD19 support the feasibility of “off‐the‐shelf” treatments against paediatric B‐cell acute lymphoblastic leukaemia (B‐ALL).

**Conclusion:**

While base editing excels in precision and functional preservation, CRISPR‐Cas9 remains preferred when complete gene knockout is desired. As off‐tumour toxicity and fratricide are addressed, the future clinical impact of these technologies is poised to expand.

## 1 Introduction

Leukaemia is a haematologic disease characterised by the uncontrolled proliferation of abnormal white blood cells in the bone marrow and peripheral blood. It is broadly classified into acute and chronic forms, with acute myeloid leukaemia (AML) and acute lymphoblastic leukaemia (ALL) representing the most aggressive subtypes [1]. Despite advancements in conventional therapies such as chemotherapy, radiotherapy and haematopoietic stem cell transplantation (HSCT), leukaemia remains a major cause of morbidity and mortality, with high relapse rates and treatment‐related toxicities limiting overall survival [2, 3]. The need for more precise and effective therapeutic strategies has driven growing interest in gene‐editing technologies as a potential cure.

**Figure 1:**
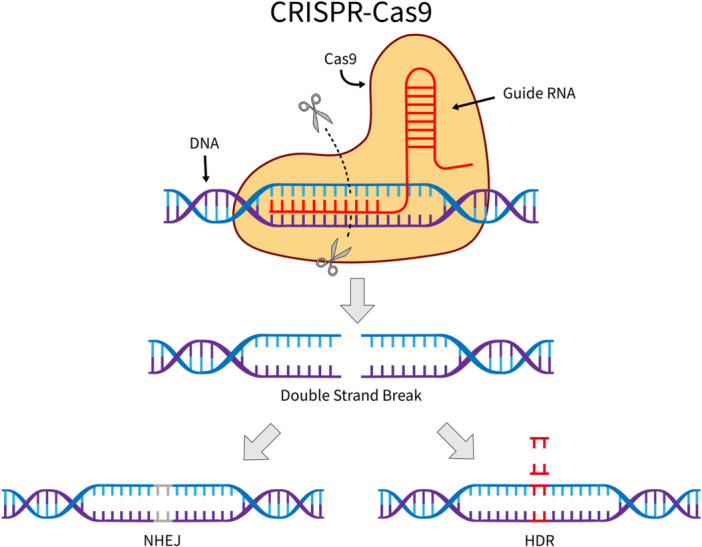
Overview of CRISPR‐Cas9. Cas9 nuclease, guided by a gRNA, introduces a double‐strand break at a complementary DNA sequence, which enables genome editing through repair pathways such as NHEJ or HDR. Grey bases represent NHEJ repair and red bases the sequence incorporated via HDR.

One of the most impactful gene‐editing tools in biomedical science is CRISPR‐Cas9 [4, 5, 6], derived from the bacterial CRISPR (Clustered Regularly Interspaced Short Palindromic Repeats) adaptive immune system [7]. CRISPR‐Cas9 uses a guide RNA (gRNA) to direct the Cas9 nuclease to a specific DNA sequence, where it induces a targeted double‐strand break (DSB). This break is subsequently repaired by cellular mechanisms, typically through non‐homologous end joining (NHEJ) or homology‐directed repair (HDR), enabling gene disruption or precise sequence modifications, respectively (Figure 1). CRISPR‐Cas9 has transformed research on leukaemia immunotherapy [8, 9]. It has been used to knock out antigens in healthy haematopoietic cells that are also expressed on malignant cells, rendering the healthy cells invisible. This strategy enables more aggressive tumour targeting while minimising collateral damage. Another application of CRISPR‐Cas9 is the editing of T cells after introduction of the chimeric antigen receptor (CAR) genes for the engineering of specialised CAR T cells [10, 11].

**Figure 2:**
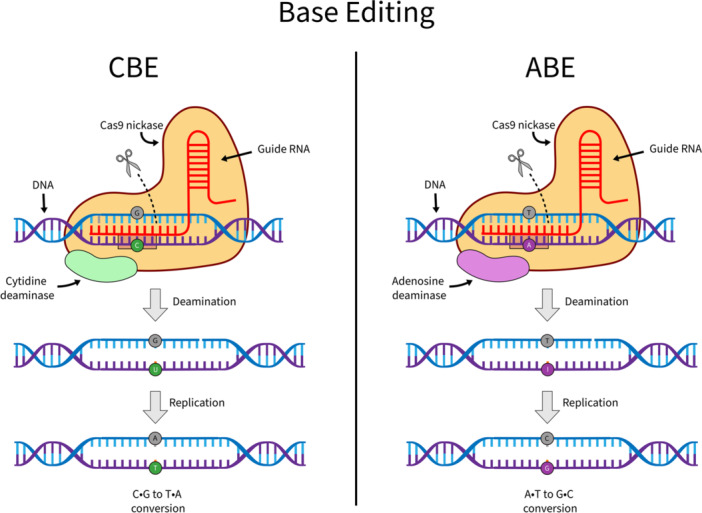
Overview of base editing. Base editors consist of a catalytically impaired nCas9 fused to a DNA deaminase, enabling the conversion of a single base without generating double‐strand breaks. CBEs deaminate C to U, read as T during replication, leading to a C•G to T•A change. ABEs deaminate A to I, read as G, producing an A•T to G•C change. The highlighted DNA region marks the deamination window.

While immunotherapy has advanced significantly through the use of CRISPR‐Cas9, this technology remains constrained by the adverse effects resulting from DSBs, including chromosomal rearrangements and unintended DNA cleavage [12, 13]. To overcome these limitations, base editing has emerged as a CRISPR‐Cas9 innovation that enables precise single‐nucleotide alterations without inducing DSBs [14, 15, 16, 17, 18]. Base editing employs a Cas9 nickase (nCas9), which introduces a single‐strand break (SSB), fused to a DNA deaminase to convert a single DNA base. Cytosine base editors (CBEs) deaminate cytosine (C) to uracil (U), which behaves like thymine (T) and, after DNA replication, results in a permanent C•G to T•A base‐pair change. Adenine base editors (ABEs) deaminate adenine (A) to inosine (I), which is interpreted as guanine (G), leading to a A•T to G•C change (Figure 2). Due to enhanced precision and reduced risk of collateral damage compared to conventional CRISPR‐Cas9 (hereafter CRISPR‐Cas9), base editing has emerged as a promising tool for the development of safer and more effective immunotherapies for leukaemia. Epitope editing represents one of its most successful applications. Epitopes are the specific sites of surface antigens that are recognised by therapeutic antibodies or CAR T cells. By modifying only the epitope, while preserving the overall antigen structure, healthy cells can evade immune attack without compromising their physiological role [19]. Despite its advantages over CRISPR‐Cas9, base editing is not without safety risks in clinical applications, including conversion of SSBs into DSBs, deaminase‐induced off‐target mutations and adverse cellular responses to the editing components [20].

Previous reviews have examined the use of CRISPR‐Cas9 for the precise engineering of CAR T cells in cancer immunotherapy [21], especially in the context of ALL [22]. Here, we highlight advances from CRISPR‐Cas9 to base editing in leukaemia immunotherapy, focussing on examples targeting CD33, CD7, CD45 and CD19, which are among the most extensively investigated antigens in the field. We provide an overview of all studies examined in Table 1, summarising key information such as target antigen, CRISPR technique and study outcomes.

**Table 1:** Summary of CRISPR‐Cas9 and base‐editing studies reviewed.

Study	Leukaemia type	Target antigen	CRISPR technique	Treatment	Outcomes	Significance
[8]	AML	CD33	CRISPR‐Cas9	CD33^KO^ HSPCs combined with CART33	86% editing efficiency; multilineage engraftment in rhesus macaques; AML clearance in engrafted mice	Proof that CRISPR‐mediated CD33^KO^ in HSPCs enables AML therapy sparing healthy myeloid cells
[9]	AML	CD33	CRISPR‐Cas9	CD33^KO^ HSPCs combined with CART33 or anti‐CD33 ADCs	90% editing efficiency; robust haematopoiesis in mice; AML clearance in a cell line‐derived mouse model	CAR T‐cell approach extended to ADCs
[23]	AML	CD33	Base editing (ABE)	HSPCs base‐edited at CD33 splice sites for anti‐CD33 immunotherapy	> 95% editing efficiency; no CD33 expression in HSPCs; normal engraftment in mice	Massively parallel base‐editing screens in HSPCs
[24]	AML	CD33, CLL‐1 and CD7	Base editing (CBE)	CART33, CARTCLL‐1 and CART7, combined with base editing of *CD52*, *CD7* and *TRBC*	Clearing of bone marrow disease in mice with combined BE CART33 and BE CARTCLL‐1 more effective than monotherapy; retention of BE CART33 anti‐leukaemia activity when co‐injected with BE CART7 in a patient‐derived xenograft model	Simultaneous use of BE CART33, BE CARTCLL‐1 and BE CART7 against heterogeneous AML
[10]	T‐ALL	CD7	CRISPR‐Cas9	CD7^KO^CART7	Anti‐leukaemic activity without fratricide in vitro and in xenografted mice	Evidence that CD7^KO^ prevents fratricide and enables generation of CD7^KO^CART7
[11]	T‐ALL	CD7	CRISPR‐Cas9	CD7^KO^TRAC^KO^CART7	Anti‐tumour activity in vitro and in vivo*;* no GvHD observed in vivo	CAR T cells without fratricide or GvHD potential
[25]	T‐ALL	CD7 and CD3	Base editing (CBE)	CART7 and CART3, combined with base editing of *CD7* and *TRBC*	Reduced chromosomal translocations between *CD7* and *TRBC* loci compared to CRISPR‐Cas9; strong cytotoxicity in T‐ALL models; no fratricide	Multiplex base editing of *CD7* and *TRBC* for combinational activity
[26]	T‐ALL	CD7	Base editing (CBE)	7CAR8 (a quadruple‐edited CAR T‐cell product targeting CD7)	No disease in mice treated with highest dose; marked reduction of leukaemia in patient‐derived xenograft models	Quadruple modification of CAR T cells without translocations or karyotypic abnormalities
[27]	T‐ALL	CD7	Base editing (CBE)	CART7, combined with base editing of *CD52*, *CD7* and *TRBC*	Minimal residual disease in child 1; fatal fungal complications in child 2; molecular remission in child 3; cytokine release syndrome and multilineage cytopenia observed	First‐in‐human application of BE CART7
[28]	T‐ALL	CD7	Base editing (CBE)	CART7, combined with base editing of CD52, CD7 and TRBC	Deep remission in 9 of 11 patients at 28 days with subsequent stem cell transplant; ongoing remission in 7 patients at 3–36 months; manageable cytokine release syndrome and rash	First phase I trial of BE CART7
[29]	AML or T‐ALL	CD45	Base editing (ABE)	CART45, combined with base editing of CD45 epitopes	AML eradicated from mice within 3 weeks of injection; treatment effective against AML, T‐ALL and B‐cell lymphoma cell lines	Epitope editing of universal antigen CD45 that preserved phosphatase function and evaded CAR recognition
[30]	AML or T‐ALL	CD45	CRISPR‐Cas9	CD45^KO^CART45 and CD45^KO^NK cells targeting CD45	In vitro elimination of PBMCs; depleted host haematopoiesis in vivo	Strategy combining HSCT preconditioning and leukaemia treatment
[31]	AML	CD45	CRISPR‐Cas9	CD45^KO^CART45	AML cells killed in vitro after 30 days of culture; no functional difference between CD45^KO^CART45 and CART45	Evidence that CD45^KO^CART45 maintain cytotoxicity even after 30‐day cultures
[32]	B‐ALL	CD19 and CD22	CRISPR‐Cas9	CTA101 (a CAR T‐cell product targeting CD19 and CD22 with disrupted *TRAC* and *CD52*)	Complete remission in 5 of 6 patients after 28 days; no dose‐limiting toxicity, GvHD, neurotoxicity or side effects; manageable cytokine release syndrome in all subjects	Support for allogeneic off‐the‐shelf treatment against R/R B‐ALL
[33]	B‐ALL	CD19	CRISPR‐Cas9	TT52CAR19 (a CAR T‐cell product targeting CD19 with disrupted *TRAC* and *CD52*)	Flow cytometry‐confirmed remission in 4 of 6 children at 28 days with stem cell transplant offered; ongoing remission in 2 children at 12 and 3 months; manageable cytokine release syndrome in all; one severe neurotoxicity	Feasibility and safety of CRISPR‐engineered immunotherapy in children with R/R B‐ALL

## 2 Engineering Safer Anti‐CD33 Therapies for AML

The earliest advances in genetic therapy for leukaemia began with CRISPR applications to knock out *CD33* in haematopoietic stem and progenitor cells (HSPCs). CD33 is a common immunotherapy target in AML, but its presence on healthy myeloid cells and HSPCs poses a risk of collateral damage. Kim et al. [8] demonstrated that CRISPR‐Cas9 could be used to selectively eliminate CD33 in human HSPCs with a 86% editing efficiency. Rhesus macaques transplanted with *CD33*‐knockout (CD33^KO^) HSPCs showed successful multilineage engraftment. The authors used anti‐CD33 CAR T cells (CART33) to eradicate AML in engrafted mice while sparing normal cells, paving the way to immunotherapy without on‐target/off‐tumour toxicity. Borot et al. [9] provided similar evidence using antibody‐drug conjugates (ADCs) in addition to CART33, injecting mice with leukaemia cells at the same time and not after CD33^KO^ HSPC engraftment. To reduce adverse effects, subsequent studies demonstrated that targeted deletion of only the V‐set domain of CD33 can prevent therapeutic binding and shield normal HSPCs [34].

Despite their significance, the studies above were limited by the potential genotoxicity intrinsic to DSBs. To mitigate this, Martin‐Rufino et al. [23] suggested a base‐editing approach. They performed an ABE screen to target all splice sites of *CD33*, identifying those eliminating gene expression. The authors achieved the same editing efficiency as CRISPR‐Cas9, with CD33 base‐edited (BE) HSPCs confirming engraftment in mice. Compared to CRISPR‐Cas9, base‐editing represented a safer alternative for AML immunotherapy and enabled the development of massively parallel screens in HSPCs.

The majority of AML blasts exhibit heterogeneous antigen expression, which limits the efficacy of single‐target CAR T‐cell therapies. Kadirkamanathan et al. [24] addressed this issue. In mice engrafted with human AML cell lines, combined BE CAR T cells targeting CD33 and CARCLL‐1 (BE CART33 and BE CARTCLL‐1) achieved greater clearance of bone marrow disease than either monotherapy. In addition, in a patient‐derived xenograft model of AML, BE CART33 retained anti‐leukaemia activity when co‐administered with BE CAR T cells targeting CD7 (BE CART7). Such strategies may enable deeper leukaemia clearance.

## 3 Redefining CD7 as a Leukaemia‐Selective Target in T‐ALL

A similar progression was observed in efforts to target CD7, a transmembrane protein expressed by T cells and natural killer (NK) cells. CD7 is also highly expressed on the surface of T‐cell ALL (T‐ALL) cells, but targeting this antigen is challenging due to fratricide among CAR T cells. Gomes‐Silva et al. [10] used CRISPR‐Cas9 to knock out *CD7* in T cells before transducing them with a CD7‐specific CAR. *CD7*‐knockout CAR T cells targeting CD7 (CD7^KO^CART7) were capable to kill T‐ALL cells *in vitro* and offered robust protection against leukaemia progression in xenografted mice, thus laying the groundwork for fratricide‐free immunotherapy. In addition to *CD7*, Cooper et al. [11] also disrupted expression of *TRAC*, which encodes the constant region of the T‐cell receptor (TCR) alpha chain. Treatment with *CD7*‐ and *TRAC*‐knockout T cells targeting CD7 (CD7^KO^TRAC^KO^CART7) prevented graft‐vs‐host disease (GvHD) in mice while keeping efficacy. This was an important step towards a strategy where allogeneic donor T cells can be engineered to target the disease.

Building on this, Georgiadis et al. [25] employed base editing on both *CD7* and *TRBC*, disrupting TCR beta chain expression and preventing the assembly of the TCR/CD3 complex. This enabled a combinational therapy with anti‐CD3 and anti‐CD7 CAR T cells (CART3 and CART7) that reduced the risks of chromosomal rearrangements compared to CRISPR‐Cas9. BE CART3 and BE CART7 showed high levels of cytotoxicity against cells expressing CD3 and CD7. They also retained potent anti‐leukaemic activities *in vivo*, confirming the fratricide‐resistant nature of the treatment. Further advancements in multiplex editing were reported by Diorio et al. [26], who developed a quadruplex‐edited CAR T‐cell product named 7CAR8. The four simultaneous edits prevented expression of CD52, CD7, PD1 and TCR. The 7CAR8 cells confirmed strong anti‐leukaemic activity both in vitro and in vivo against T‐ALL cell line models, prolonging the survival of six patient‐derived xenograft mouse models.

The research above culminated in the pioneering clinical application reported by Chiesa et al. [27], who treated three paediatric patients with relapsed T‐ALL using BE CART7. To avoid lymphodepleting serotherapy, fratricide and GvHD, the authors edited *CD52*, *CD7* and *TRBC*, respectively. Despite several reported adverse effects, including cytokine release syndrome and multilineage cytopenia, these interim results suggested an anti‐leukaemic effect of allogeneic BE CART7, with one of the three patients achieving complete molecular remission within 28 days and undergoing stem cell transplant. This anti‐leukaemic activity was subsequently confirmed by the full phase I trial, which reported consistent efficacy and safety outcome [28].

## 4 Targeting CD45 for Universal CAR T‐Cell Therapy

As CRISPR technologies matured, a key focus shifted from patient‐specific therapies towards developing universal treatments that can be applied across diverse leukaemia subtypes [35]. Wellhausen et al. [29] pioneered the epitope base‐editing of CD45, a receptor‐type protein tyrosine phosphatase expressed on the surface of most normal and malignant haematopoietic cells. Remarkably, BE CAR T cells targeting CD45 (BE CART45) were able to expand. Opposite to CRISPR‐Cas9, BE CART45 preserved their function, clearing engrafted mice from AML within 3 weeks of injection. In addition, BE CART45 were effective at eliminating AML, T‐ALL and B‐cell lymphoma cell lines, while all other engineered CAR T cells investigated could only attack their lineage‐specific tumour cells. This approach paved the way for future pan‐haematologic CAR T‐cell immunotherapy.

While base editing has demonstrated clear advantages in terms of safety, precision and editing efficacy, recent studies suggest that CRISPR‐Cas9 still holds strategic value for selective knockout strategies. For example, Stepanova et al. [30] demonstrated that CRISPR‐Cas9 can be used to disrupt CD45 expression in CART45. The *CD45*‐knockout (CD45^KO^)CART45 showed more effective killing of T‐ALL, AML and Mantle cell lymphoma cells than CART45. The authors confirmed the potent anti‐tumour activity of CD45^KO^CART45 in a mouse xenograft model of AML. Both CD45^KO^CART45 and CD45^KO^NK cells targeting CD45 eliminated peripheral blood mononuclear cells (PBMCs) in vitro, with CD45^KO^CART45 capable of depleting haematopoietic cells in humanised mice. In line with the results above, Harfmann et al. [31] found that CD45^KO^CART45 are not subject to fratricide and maintain their functionality in vitro. Together, the last two studies challenge the notion that base editing is better than CRISPR‐Cas9 in all clinical contexts, suggesting that the two tools should be viewed as complementary rather than competing.

## 5 Enabling Off‐the‐Shelf Therapy for B‐ALL

Although autologous CD19‐specific CAR T cells improve outcomes for refractory and relapsed (R/R) B‐cell ALL (B‐ALL) [36], their personalised nature poses logistical, manufacturing and cost challenges. Donor‐derived anti‐CD19 CAR T cells could enable rapid and off‐the‐shelf therapy for multiple B‐ALL patients [37]. Building on a previous approach using transcription activator‐like effector nucleases (TALENs) to engineer CD19‐targeted CAR T cells [38], Hu et al. [32] developed CTA101, a CRISPR‐Cas9‐based product targeting both CD19 and CD22. They disrupted *TRAC* and *CD52*, respectively, to prevent GvHD and render the CAR T cells resistant to the anti‐CD52 monoclonal antibody used during lymphodepletion. The phase I clinical trial showed that CTA101 was tolerated, with a manageable cytokine release syndrome occurring in all six patients. By day 28, a complete remission rate was achieved in five of six patients. In another phase I clinical trial [33], adopted TT52CAR19, a CD19‐directed CAR T‐cell product engineered with CRISPR‐Cas9‐mediated disruption of *TRAC* and *CD52*. Four of six patients treated with TT52CAR19 achieved flow cytometric‐confirmed remission 28 days after infusion and were offered allogeneic stem cell transplantation. Toxicities were controlled, with one child developing grade IV neurotoxicity that required treatment. Together, the two trials highlight the potential of off‐the‐shelf allogeneic CAR T cells targeting CD19 as a promising alternative to autologous therapy for B‐ALL.

## 6 Conclusions and Prospects

CRISPR genome editing has opened a new chapter in the treatment of leukaemia. Today, technologies such as CRISPR‐Cas9 and base editing enable precise modifications of key target antigens, allowing fine‐tuning of their expression without compromising any essential cellular function. As a result, major limitations of conventional CAR T‐cell therapy, including on‐target/off‐tumour toxicity and fratricide, can be addressed.

We note that most supporting evidence to date comes from in vitro or murine studies, which provides limited data about long‐term safety and efficacy in humans. However, it is significant that in less than a decade the field has advanced from conceptual CRISPR‐Cas9 groundwork to the development of safer base‐editing strategies capable of achieving molecular remission. This rapid momentum suggests that clinical validation is on the horizon. As CRISPR technologies continue to mature, they are set to revolutionise how we treat leukaemia and other diseases.

## Author Contributions

Dejin Rai carried out the project under the supervision of Umberto Terranova. Both authors wrote the manuscript. Both authors have read and approved the final version of the manuscript. Umberto Terranova takes complete responsibility for the integrity and accuracy of the review.

## Disclosure

The lead author Umberto Terranova affirms that this manuscript is an honest, accurate, and transparent account of the study being reported; that no important aspects of the study have been omitted; and that any discrepancies from the study as planned (and, if relevant, registered) have been explained.

## Ethics Statement

This review was not subject to ethical approval as only previously published data was reported.

## Conflicts of Interest

The authors declare no conflicts of interest.

## Data Availability

Data sharing not applicable to this article as no datasets were generated or analysed during the current study.
